# Discontinuation of standard first-line antiretroviral therapy in a cohort of 1434 Malawian children

**DOI:** 10.1186/1758-2652-13-31

**Published:** 2010-08-06

**Authors:** W Chris Buck, Mark M Kabue, Peter N Kazembe, Mark W Kline

**Affiliations:** 1 Baylor College of Medicine International Pediatric AIDS Initiative, Houston, Texas, USA; 2 Baylor College of Medicine-Abbott Fund Children's Clinical Centre of Excellence - Malawi; 3 Department of Pediatrics, Baylor College of Medicine, and Texas Children's Hospital, Houston, Texas, USA

## Abstract

The standard first-line antiretroviral (ART) regimen in Malawi for both adults and children is a fixed-dose combination tablet containing stavudine (d4T), lamivudine (3TC) and nevirapine (NVP). This regimen has been shown to yield satisfactory virologic and immunologic outcomes in children. Published studies have described insights into discontinuation of first-line regimen and toxicities of ART in adults, but similar studies in paediatric populations are lacking.

A retrospective cohort study was undertaken to assess reasons for discontinuation of the standard first-line ART regimen (d4T/3TC/NVP) in a paediatric population. In total, 1434 patients met eligibility criteria and were included. The cohort had mean and median age at ART initiation of 4.7 years and 2.9 years, respectively (range: 0.1 months-18.7 years). The gender distribution was 47% female and 53% male. Median follow-up time on ART was 1.8 years (range: 2 weeks-3.9 years). A majority (96.2%) of patients were on the standard first-line ART regimen, while 3.8% (54) were on a different regimen. Twenty-eight patients (2.0%) were on an alternative first-line regimen due to toxicities, 22 patients (1.5%) were on a second-line regimen due to ART failure, and four patients (0.3%) were on a non-standard regimen for other clinical reasons.

Of the 28 patients who experienced toxicities requiring ART regimen change, 60.7% (17) were caused by NVP, 39.3% (11) by d4T, and none by 3TC. The median time from first-line ART initiation to alternative first-line ART was two months (range: 10 days-28.1 months); 60.7% of patients on alternative first-line ART were male. Average time on ART until switch to second-line ART regimen was 16.3 months (SD: 9.3 months). The probability of failure after one year on first-line regimen was 1.6% (95% CI: 0.9-2.6).

There was no compelling evidence in this cohort, representing approximately 10% of all children on ART in Malawi, to support changing the standard paediatric first-line regimen based on early toxicities or failure. However, experience from the national adult cohort, longer term follow up of the paediatric cohort in this study, emerging data on resistance after single-dose NVP containing mother to child transmission antiretroviral prophylaxis, and new 2009 World Health Organization ART recommendations may influence national policy change to a different first-line regimen.

## Findings

The standard first-line antiretroviral (ART) regimen in Malawi for both adults and children is a fixed-dose combination tablet containing stavudine (d4T), lamivudine (3TC) and nevirapine (NVP). This regimen is cheap and convenient, especially where there are limited affordable alternatives for children in resource-limited countries like Malawi. NVP is dosed once daily for the first two weeks of ART using separate d4T/3TC and NVP tablets, and then increased to twice daily dosing using d4T/3TC/NVP fixed-dose combination tablets if well tolerated.

Children are dosed according to locally-developed weight-band dosing tables using fractions of adult Triomune 30 tablets. This approach has been shown to result in satisfactory virologic and immunologic benefit to children [1]. However, there are concerns about NVP drug levels in children on fractionated adult tablets [2]. There is also evidence that single-dose NVP (sdNVP)-containing mother to child transmission (MTCT) antiretroviral (ARV) prophylaxis regimens increase the risk of resistance and failure of NVP-based ART regimens in children [3]. During the time of this study, all children received split adult Triomune 30 tablets as the paediatric formulation; Triomune Baby was introduced at a national level in 2009, after our study period. In addition, there were no provisions for a protease inhibitor-based first-line regimen for infants with sdNVP MTCT ARV prophylaxis exposure in the national ART guidelines.

The national ART programme offers additional ART regimens when d4T/3TC/NVP is no longer a tolerable or effective regimen. In the case of adverse drug reactions or toxicities to the first-line regimen, alternative first-line regimens are available, which replace d4T with zidovudine (AZT), and NVP with efavirenz (EFZ). In cases of ART failure, which can be diagnosed clinically, immunologically or virologically depending on the setting, second-line regimens are available. The adult second-line regimen is AZT, 3TC, tenofovir and lopinavir/ritonavir, while the paediatric second-line regimen is abacavir, didanosine and lopinavir/ritonavir.

The Malawi Ministry of Health is investigating a potential change of the national standard first-line regimen, in part due to concerns about the high prevalence of d4T-related toxicities, particularly peripheral neuropathy in adults, in line with guidelines released World Health Organization (WHO) in late 2009 on preferred first-line ART regimens [4]. Published studies have described insights into discontinuation of first-line regimen and toxicities to ART in adults [5], but similar studies in paediatric populations are lacking [6,7].

An analysis of retrospectively collected cohort data was undertaken to assess the reasons for discontinuation of standard first-line ART in a cohort of children at the Baylor College of Medicine-Abbott Fund Children's Clinical Centre of Excellence-Malawi as a step towards assisting the Ministry of Health in making an informed decision based on locally available data. Our paediatric HIV care programme operates from the capital city, Lilongwe, and has enrolled more than 4500 infants and children since its inception in 2004. As of July 2009, there was an active caseload of 2330 HIV-infected and exposed patients, 1444 of whom were on ART. By the end of 2008, our patient population represented approximately 10% of all children ever initiated on ART in Malawi [8].

We investigated the discontinuation of standard first-line ART as our primary outcome. Toxicities were defined as adverse events reported after ART initiation, which were confirmed clinically or with laboratory tests when available. ART failure was identified clinically, immunologically and virologically using national ART and WHO guidelines.

Eligibility criteria for the study were patients aged 19 years or younger, with at least two clinic visits, who were ART-naïve at the time of standard first-line initiation (excluding MTCT ARV prophylaxis exposure), and who had enrolled between November 2004 and October 2008, either at our clinic or at its predecessor clinic at Kamuzu Central Hospital in Lilongwe. All patients had records in our electronic medical record (EMR) which were de-identified and subsequently reviewed. Patients on an ART regimen other than the standard first-line regimen were identified through the EMR and confirmed by cross checking with our paper-based national ART registers. It is not possible to reliably query our EMR for patients who had ART toxicities or first-line failure that did not result in an ART regimen change. For this reason, we used discontinuation of the first-line regimen as a proxy for toxicity and failure. Data were analyzed using STATA version 9 (STATA Corporation, College Station, Texas, USA).

A total of 1434 patients met eligibility criteria and were included in this review. Fewer than 15 patients had stopped ART for poor adherence or social concerns at the time of the analysis and were not included in this review as they were expected to resume the standard first-line regimen at the time of re-initiation. The cohort had mean and median ages at ART initiation of 4.7 years and 2.9 years, respectively, with a range of 0.1 months to 18.7 years. The gender distribution was 47% female and 53% male. The median follow-up time on ART was 1.8 years with a range of two weeks to 3.9 years.

A majority of patients (96.2%) were on the standard first-line ART regimen, while 3.8% (54) were on a different regimen: 28 patients (2.0%) were on an alternative first-line regimen due to toxicities, 22 patients (1.5%) were on a second-line regimen due to failure, and four patients (0.3%) were on a non-standard regimen for other clinical reasons. Of the 28 patients with toxicities requiring ART regimen change, 60.7% (17) were related to NVP, 39.3% (11) were related to d4T, and none were related to 3TC, as shown in Table 1.

**Table 1:** Reasons for discontinuation of standard first-line ART regimen

Reason for discontinuation of **standard first-line ART (d4T/3TC/NVP) **^**a**^	Number of patients (male/female)	Proportion of total cohort of 1434 patients (%)
**Toxicity**	**28 (17/11)**	**1.95%**

• Nevirapine - rash (unknown WHO grade)	1 (0/1)	0.07%

• Nevirapine - rash (WHO grade II)	5 (3/2)	0.35%

• Nevirapine - rash (WHO grade III)	4 (2/2)	0.28%

• Nevirapine - Stevens-Johnson syndrome (WHO grade IV) ^b^	5 (4/1)	0.35%

• Nevirapine - hepatitis (WHO grade IV) ^c^	2 (2/0)	0.14%

**• Nevirapine - total**	**17 (11/6)**	**1.19%**

• Stavudine - pancreatitis (WHO grade III)	3 (2/1)	0.21%

• Stavudine - pancreatitis (WHO grade IV)	2 (1/1)	0.14%

• Stavudine - lactic acidosis (unknown WHO grade)	1 (1/0)	0.07%

• Stavudine - lactic acidosis (WHO grade III)	3(1/2)	0.21%

• Stavudine - peripheral neuropathy (unknown WHO grade)	1 (0/1)	0.07%

• Stavudine toxicity - peripheral neuropathy (WHO grade III)	1 (1/0)	0.07%

**• Stavudine - total**	**11 (6/5)**	**0.76%**

**First-line failure ^d^**	**22 (16/6)**	**1.53%**

**Other ^e^**	**4 (3/1)**	**0.28%**

^a^Toxicity grading per 2006 WHO guidelines (I-mild, II-moderate, III-severe, IV-severe, potentially life-threatening).

^b^One patient died from drug-induced SJS.

^c^One patient had a negative Hepatitis B surface antigen test documented. Serologies for other viral hepatitides are not available in Malawi. Neither patient was on concurrent TB treatment.

^d^All patients in our cohort had confirmed virologic failure on first-line ART.

^e^Four Kaposi's sarcoma patients had been placed on a protease inhibitor-based regimen for possible antiangiogenic effects.

The median time to substitution of alternative first-line ART was two months, with a range of 10 days to 28.1 months. Of the patients on alternative first-line ART, 60.7% were male. Average time on ART until switch to second-line ART regimen was 16.3 months (SD: 9.3 months). The probability of failure after one year on the first-line regimen was 1.6% (95% CI: 0.9-2.6).

This retrospective cohort review shows that the Malawian standard first-line ART regimen of d4T/3TC/NVP fixed-dose combination resulted in limited early toxicities requiring ART regimen change in this paediatric population. It also shows that early first-line ART failure requiring switch to second-line ART was not commonly diagnosed in our cohort.

There are limitations of this retrospective review that should be mentioned. First, due to the study design, there are certainly patients in this large cohort with toxicities that were not captured, either because the patients' adverse reactions were not severe enough to warrant a regimen change or because they were never diagnosed. This is of particular concern with respect to lipodystrophy. In addition, as per national guidelines, patients did not get routine laboratory monitoring for ART toxicities, such as liver function tests, pancreatic enzymes or other chemistry investigations. This approach of no routine laboratory monitoring has been validated as cost effective and safe in African settings, but likely results in under-diagnosis of sub-clinical laboratory toxicities [9].

Another limitation of our study design is that it only captured patients who had been switched to second-line ART for confirmed failure. Our patients do get biannual CD4 testing, but routine viral load testing is not done at our clinic, or anywhere in the Malawi national ART programme. As a result, first-line failure is almost certainly under-represented here as patients have to present with later-onset clinical or immunologic failure before viral load tests are ordered.

A final limitation is that the median time on ART was relatively short at only 1.8 years, as the national ART programme only began including ART for children in the public health facilities in 2004. Many metabolic complications from longer term d4T treatment, including peripheral neuropathy, lactic acidosis and lipodystrophy, might not have presented within this limited timeframe. A similar analysis will need to be repeated in the future to evaluate metabolic complications after patients have been on ART for longer periods.

In conclusion, there was no compelling evidence in this cohort, representing approximately 10% of all children on ART in Malawi, to support changing the standard Malawian first-line regimen of d4T/3TC/NVP based on early toxicities or failure. However, experience from the adult cohort, longer term follow up of the paediatric cohort in this study, emerging data on NVP resistance after sdNVP-containing MTCT ARV prophylaxis, and new 2009 WHO ART recommendations may influence national policy change to a different first-line regimen.

## Competing interests

The authors declare that they have no competing interests.

## Authors' contributions

WCB participated in the design of the study, data collection, and writing of the manuscript. MMK participated in the design of the study, data collection, data analysis, and writing of the manuscript. PNK participated in the design of the study, and reviewing the manuscript. MWK participated in revising the manuscript, and giving final approval of the version to be published.

All authors have read and approved the final manuscript.

## References

[B1] Chokephaibulkit K Plipat N Cressey TR Frederix K Phongsamart W Capparelli E Kolladarungkri T Vanprapar N Pharmacokinetics of nevirapine in HIV-infected children receiving an adult fixed-dose combination of stavudine, lamivudine and nevirapine AIDS 2005 13 1495 1499 10.1097/01.aids.0000183625.97170.59 16135903

[B2] Ellis J L'homme R Ewings F Mulenga V Bell F Chileshe R Molyneux E Abernathy J van Oosterhout J Chintu C Walker S Gibb D Burger D Nevirapine concentrations in HIV-infected children treated with divided fixed-dose combination antiretroviral tablets in Malawi and Zambia Antiviral Therapy 2007 13 253 260 17503667

[B3] Palumbo P Violari A Lindsey J Hughes M Jean-Philippe P Mofenson L Purdue L Eshleman S Nevirapine vs. lopinavir-ritonavir-based antiretroviral therapy (ART) in single dose nevirapine (sdNVP)-exposed HIV infected infants: preliminary results from the IMPAACT P1060 trial HIV Pediatrics Cape Town, abstract LBPEB12, July 2009

[B4] World Health Organization Rapid advice: antiretroviral therapy for HIV infection in adults and adolescents. Electronic version 2009 http://www.who.int/hiv/pub/arv/rapid_advice_art.pdf

[B5] d'Arminio Monforte A Lepri AC Rezza G Pezzotti P Antinori A Phillips AN Angarano G Colangeli V De Luca A Ippolito G Caggese L Soscia F Filice G Gritti F Narciso P Tirelli U Moroni M Insights into the reasons for discontinuation of the first highly active antiretroviral therapy (HAART) regimen in a cohort of antiretroviral naïve patients. I.CO.N.A. Study Group. Italian Cohort of Antiretroviral-Naïve Patients AIDS 2000 13 Suppl 5 499 507 10.1097/00002030-200003310-00005 10780712

[B6] Gil AC Lorenzetti R Mendes GB Morcillo AM Toro AA Silva MT Vilela MM Hepatotoxicity in HIV-infected children and adolescents on antiretroviral therapy Sao Paulo Med J 2007 13 205 209 10.1590/S1516-31802007000400002 17992389 PMC11020542

[B7] McComsey G Leonard E Metabolic complications of HIV therapy in children AIDS 2004 13 1753 1768 10.1097/00002030-200409030-00004 15316336

[B8] Malawi Ministry of Health, HIV Unit Treatment of AIDS. A Five-Year Plan for the Provision of Antiretroviral Therapy and Good Management of HIV-related Diseases to HIV-infected Patients in Malawi 2006-2010 http://www.hivunitmohmw.org/uploads/Main/ARV-ScaleUpPlan_2006-2010.pdf

[B9] Dart Trial Team Routine versus clinically driven laboratory monitoring of HIV antiretroviral therapy in Africa (DART): a randomised non-inferiority trial Lancet 2010 13 123 131 10.1016/S0140-6736(09)62067-5 PMC2805723 20004464

